# The ethical value of consulting community members in non-emergency trials conducted with waivers of informed consent for research

**DOI:** 10.1177/17407745241259360

**Published:** 2024-06-25

**Authors:** Emily A Largent, Steven Joffe, Neal W Dickert, Stephanie R Morain

**Affiliations:** 1 Department of Medical Ethics and Health Policy, Perelman School of Medicine, University of Pennsylvania, Philadelphia, PA, USA; 2 Department of Medicine, Division of Cardiology, Emory University School of Medicine, Atlanta, GA, USA; 3 Berman Institute of Bioethics, Johns Hopkins University, Baltimore, MD, USA

**Keywords:** Consent, ethics, waivers, pragmatic trials

## Abstract

There is growing interest in using embedded research methods, particularly pragmatic clinical trials, to address well-known evidentiary shortcomings afflicting the health care system. Reviews of pragmatic clinical trials published between 2014 and 2019 found that 8.8% were conducted with waivers of informed consent; furthermore, the number of trials where consent is *not* obtained is increasing with time. From a regulatory perspective, waivers of informed consent are permissible when certain conditions are met, including that the study involves no more than minimal risk, that it could not practicably be carried out without a waiver, and that waiving consent does not violate participants’ rights and welfare. Nevertheless, when research is conducted with a waiver of consent, several ethical challenges arise. We must consider how to: address empirical evidence showing that patients and members of the public generally prefer prospective consent, demonstrate respect for persons using tools other than consent, promote public trust and investigator integrity, and ensure an adequate level of participant protections. In this article, we use examples drawn from real pragmatic clinical trials to argue that prospective consultation with representatives of the target study population can address, or at least mitigate, many of the ethical challenges posed by waivers of informed consent. We also consider what consultation might involve to illustrate its feasibility and address potential objections.

The practice of requiring researchers to obtain informed consent from research participants is grounded in the ethical principle of respect for persons.^1^ The process of informed consent gives prospective participants key information about a study so that they can determine for themselves whether participation is consistent with their values and preferences. Although prospective informed consent for research (“traditional consent”) is typically required, there are exceptions.

These exceptions are found in regulations and influential guidance documents. In the United States, the Federal Policy for the Protection of Human Subjects, or “Common Rule,” permits waivers or alterations of informed consent when a study meets certain conditions (Table 1). US Food and Drug Administration guidance supports alterations and waivers under similar conditions.^2^ The Ottawa Statement on the Ethical Design and Conduct of Cluster Randomized Trials (“Ottawa Statement”) permits a waiver or alteration of consent requirements when the research is not feasible without a waiver or alteration and the study interventions pose no more than minimal risk.^3^ Guideline 10 of the Council for International Organizations of Medical Sciences *International Ethical Guidelines for Health-Related Research Involving Humans* also allows for modifications or waivers of informed consent but adds a third condition to the two conditions found in the Ottawa Statement: the research has important social value.^4^ Institutional review boards (IRBs) or research ethics committees (henceforth, “IRBs” for brevity) are tasked with ensuring that the conditions for a waiver of traditional consent are met.

**Table 1.** Common Rule (45 CFR §46.116(f)(3)) requirements for waivers and alterations.

In order for an IRB to waive or alter consent as described in this subsection, the IRB must find and document that: (i) The research involves no more than minimal risk to the subjects (ii) The research could not practicably be carried out without the requested waiver or alteration (iii) If the research involves using identifiable private information or identifiable biospecimens, the research could not practicably be carried out without using such information or biospecimens in an identifiable format (iv) The waiver or alteration will not adversely affect the rights and welfare of the subjects (v) Whenever appropriate, the subjects or legally authorized representatives will be provided with additional pertinent information after participation

Notably, neither the regulations nor the guidance documents distinguish between alterations of traditional consent, on one hand, and waivers of traditional consent, on the other. We believe that both alterations and waivers are justifiable but recognize these approaches are ethically distinct.^5,6^ Alterations of consent can encompass a broad range of options—from opt-out approaches to shortened consent forms—that generally afford prospective participants an opportunity to determine whether or not to enroll.^7^ Waiving consent means that there is no requirement for investigators to inform participants they are in a research study; participants will likely be unaware that they are in research and will have no opportunity to choose participation. We therefore focus on waivers of traditional consent here.

Waivers are a common feature of pragmatic clinical trials (PCTs), though pragmatism is neither a necessary nor sufficient justification for waiving consent. Because PCTs—which are generally embedded into real-world clinical practice settings and capitalize on existing clinical data sources for outcomes assessment—can be executed more quickly and efficiently than traditional clinical trials and have the potential address well-known evidentiary gaps, they have garnered substantial support from funders and are conducted with growing frequency. A review of PCTs published between 2014 and 2019 found, consistent with other estimates, that 8.8% were conducted with a waiver of traditional consent for some or all aspects of the trial.^8,9^ Moreover, the number of PCTs in which consent is *not* obtained is increasing with time.^8^ These trends have drawn renewed attention to ethical issues surrounding waivers of traditional consent.

When reviewing a PCT, IRBs often grant waivers because investigators are comparing existing or approved interventions, each of which is consistent with the standard of care, and the accompanying research procedures, such as records review to ascertain patient outcomes, introduce minimal risk. Moreover, many PCTs employ cluster randomization (e.g. in which the unit of randomization is a clinical site or operating unit rather than an individual) or other design elements that create obstacles to, or even preclude, offering individual patient-participants choices about intervention assignment or obtaining traditional consent.^7^ The appropriateness of a waiver for any particular study may be a matter of reasonable debate. Thus, there are compelling reasons to employ additional safeguards to ensure both the appropriate use of waivers of consent and the ethical design of studies that propose to include such waivers.

Here, we suggest that early community consultation—in which investigators discuss their proposed research with and seek feedback from representatives from the community of potential participants—may be a valuable tool to address four challenges of conducting PCTs with a waiver of traditional consent. These challenges are: (1) addressing empirical evidence showing that patients and members of the public generally prefer prospective consent, (2) demonstrating respect for persons using tools other than consent, (3) promoting public trust and investigator integrity, and (4) ensuring adequate protections against risk and burden. Though the idea of community consultation is not itself novel, a robust ethical argument for its importance in the PCT context has yet to be offered.

Before making this argument, two preliminaries are in order. First, while clinicians are appropriately considered research subjects in many PCTs, we focus our analysis on consultation with communities of potential *patient*-participants. Second, while focusing on investigators and patient-participants, we think the benefits of consultation will also redound to others, including the institutions that conduct PCTs and those that sponsor them.

## Four ethical challenges of waiving informed consent

### Responding to patient and public preferences for prospective consent

In recent years, researchers have explored the perspectives of diverse stakeholders, particularly patients and members of the public, about approaches to informed consent, including waivers. Across these studies, most respondents prefer prospective approaches—including traditional consent or alterations of consent—to waivers.^6^ Many respondents, however, find waiving consent acceptable once they understand the tradeoffs, especially that socially valuable research could not otherwise be conducted.^10,11^

In the absence of an interactive process that facilitates reflective deliberation, it can be difficult for investigators to know whether potential participants might accept a waiver of traditional consent for any particular PCT. Early community consultation is a means of gaining insights into how community members understand and weigh the relevant tradeoffs for a given study, while also serving to increase general awareness of the importance of research and the potential utility of waiving consent to conduct socially valuable studies. In some cases, insights from consultation might build confidence in the legitimacy and acceptability of conducting the proposed PCT with a waiver of traditional consent. In other cases, consultation might lead investigators to modify the PCT design so as to enhance acceptance of the waiver or to conduct the trial using a prospective approach, whether traditional consent or an alteration, rather than a waiver.

A consultative process may be particularly important when studies enroll members of vulnerable or marginalized groups, as negative past and ongoing experiences with research and the health care system have engendered understandable distrust.^12^ While the empirical evidence is limited, extant data suggest that members of minoritized groups may view the practice of waiving consent less favorably than others.^6^ Consider that, in the course of a discussion about conducting PCTs under waivers of traditional consent, a group of persons living with dementia and care partners for persons living with dementia (“IMPACT Lived Experience Panel”) reflected on their own experiences of stigma and discrimination in the health care system.^13^ Some spoke to their intersecting identities: for example, being a Black woman with dementia meant exposure both to racism and also to the public stigma of Alzheimer’s disease. Though nearly all IMPACT Lived Experience Panel members felt that waivers of consent could be appropriate at times, they stressed the importance of research and researchers being trustworthy. For them, that meant, in part, that there should be consultation with people living with dementia and their care partners to identify research questions that were important to their community and that consultation should continue throughout the design and conduct of research, so that, no PCTs were conducted “about us without us.”^13^

Though it is an empirical question that should be tested in diverse populations, we speculate consultation might reassure those who ultimately are enrolled in the PCT using a waiver. If an investigator can tell community members and enrolled participants—who might, for instance, only learn about their participation *after* a study is completed through notification processes—that people similar to them reviewed and endorsed the study *before* it started and found waiving consent reasonable given the tradeoffs, enrolled participants might be more accepting of the decision to forego prospective consent.

One might ask whether the ability to notify enrolled participants suggests that prospective consent was in fact possible, but there are plenty of circumstances in which a waiver is necessary though notification is feasible. For instance, a waiver might be needed to address concerns about bias, but enrolled participants can learn about the intervention or research procedures later (e.g. after delivery of the intervention or ascertainment of outcomes) when bias is no longer a concern. Or, there may be situations in which investigators can notify participants at the outset but cannot seek traditional consent or allow opt-out. For example, the Study to Understand Nighttime Staffing Effectiveness in a Tertiary Care Intensive Care Unit (SUNSET-ICU) compared two common methods of nighttime staffing: in-hospital intensivists *and* medical residents or in-hospital medical residents only.^14^ Blocks of seven consecutive nights were randomly assigned to one staffing method or the other. SUNSET-ICU was conducted using a waiver, with a key reason being that patient assignment needed to be complete (i.e. all patients had an in-hospital intensivist or not).

### Demonstrating respect for persons using tools other than consent

Waiving traditional consent requires us to reflect on what respect for persons demands.^15^ The informed consent process is rooted in the principle of respect for persons, often understood as synonymous with respect for their autonomy.^16^ But consent—or the opportunity for autonomous decision-making—is not the only instantiation of respect.^17^ It is also important for investigators to act in ways that demonstrate respect for participants and make them feel respected.^18^

Consultation has intrinsic value. The act of engaging in consultation before conducting a study shows that investigators respect their future participants and care about their wellbeing. It also demonstrates respect for the community in which the study will be conducted. This value is higher the more substantive and less performative the consultation is.

In addition, consultation has instrumental value. First, when an investigator speaks with community members about a proposed study, consultation may demonstrate respect for the community as well as future participants by approximating some of the functions of traditional consent.^18^ Consultation requires provision of a public justification for the study. By sharing reasons for decisions (in ways that are clearly explained and free from jargon), such justification can promote legitimacy by making the rationale for conducting the study, including the use of a waiver, accessible to those whose interests may be affected by the research.^19^

Second, what investigators learn from community members in the course of consultation may aid them in making participants feel respected by giving them a better understanding of the community’s values, preferences, and interests and how to accommodate them. For instance, consultation may inform or identify ways to serve some of the functions of traditional consent. It may underscore the importance, which investigators often underestimate, of notifying participants that research is occurring or has occurred. In studies where contemporaneous notification is possible, like SUNSET-ICU, investigators can learn from the community how best to talk about an ongoing trial, including how best to address the waiver and community consultation process. Furthermore, consultation can help investigators develop strategies for communicating with the individuals ultimately enrolled without prospective consent after the study is complete as well as clarify when such communication is desirable and outline participants’ informational needs.

Consider the cluster-randomized Comprehensive Post-Acute Stroke Services (COMPASS) trial, which evaluated the comparative effectiveness of a patient-centered, post-acute stroke intervention versus usual-care control.^20^ Through consultation with former stroke patients and care partners, the COMPASS team tailored study notification to best match the preferences and informational needs of potential participants and their families. In consultation, patient-stakeholders emphasized that the study notification handout should not imply usual care was inferior to the intervention, expressing concern that such a message could interfere with recovery. Patient-stakeholders also provided feedback to promote comprehension by individuals with differing health literacy levels. Ultimately, the patient handout was “iteratively revised by patient-stakeholders until it satisfied their concerns and met all IRB requirements.”^20^ These improvements to the study’s communication plans were the direct result of the investigators’ preparatory consultations with individuals who could approximate the perspectives of future study participants.

### Promoting public trust and investigator integrity

When traditional consent is waived, there is a loss of accountability, which risks undermining public trust by giving the impression that research is being designed and conducted behind closed doors. Individuals beyond those enrolled in PCTs might be concerned about the use of waivers, but may feel differently if they knew members of the relevant communities were engaged in decision-making about the trial and the use of a waiver. This concern for public trust may be especially strong for PCTs, given prior research documenting that prospective participants often misunderstand key features of these studies, such as assuming that research always involves testing investigational medications, or that it necessarily involves substantial burdens for participants—concerns that might make individuals especially leery of consent being waived.^21,22^ Experience suggests that, when participants and the public know that investigators have presented their research plans to members of stakeholder communities and genuinely sought feedback, they may be more likely to trust that the research was designed and conducted with participants’ interests and perspectives in mind.^23^

Consultation may also promote investigator accountability, and therefore investigator integrity, by requiring investigators to engage with the community. Investigators who know that they will be required to describe and justify their study to members of the community in which they propose to conduct their research may be less likely to design studies that cannot pass the test of publicity. Of course, transparency is necessary but may not be sufficient to produce accountability, and it is worth studying the conditions under which transparency does lead to accountability.^24^ Such studies can and should inform the design and implementation of consultation.

### Enhancing protections against risk and reducing burden

Asking prospective participants for traditional consent promotes their welfare interests by allowing them to consider what constitutes burdens or harms to them.^18^ When research is conducted with a waiver, investigators might design studies with unacceptable features—ones they and the reviewing IRB fail to catch—and never get the feedback that might otherwise come from low participation and high refusal rates, as well as from conversations with those who do and don’t choose to enroll. This is a lost opportunity. Consultation has instrumental value if it helps investigators understand how the proposed intervention could affect participants’ welfare. Feedback from individuals who are similar to those who might be eligible for the trial could, for example, surface issues and suggest modifications. In this way, consultation might serve a similar signaling function as informed consent by letting investigators know that a study (or a particular element of a study) is or is not acceptable to the target study population. This can reduce burden on participants and, in some cases, improve the value and rigor of the study, which is also important to ethical research.

The experience of the Time to Reduce Mortality in End Stage Renal Disease (TiME) trial team is suggestive of how consultation might help investigators prospectively identify unacceptable or undesirable aspects of their study. This cluster-randomized, pragmatic trial evaluated the effects of extended duration dialysis sessions versus usual care.^25^ The unit of randomization was the dialysis facility; the length of participants’ dialysis sessions was determined by the facility in which they received their care. When a new patient initiated maintenance dialysis, facilities randomized to the intervention conducted sessions of 4.25 h or longer, whereas facilities randomized to usual care had no specified session duration (historical data suggested that the average would be 3.5 h). The trial was conducted with a waiver of consent, though patients were given written information about the trial. Ultimately, however, the trial was stopped early due to poor adherence. While the TiME trial team noted several reasons for this, including treating nephrologists’ perception that longer sessions were not needed, the one the team deemed most important was patient-participants’ reluctance to have longer dialysis sessions than many other patients at the same dialysis facility. Looking back, the study team noted that greater prospective consultation might have led to modifications in design, intervention, and trial-related communication that could have resulted in better adherence and a more informative trial.

In addition to affording investigators important insights, consultation may also benefit IRBs. IRBs are charged with determining whether or not the conditions for a waiver obtain. While the appropriateness of waiving traditional consent is relatively straightforward for some PCTs, there are open questions and reasonable debates about others. As a result, there is heterogeneity in IRBs’ determinations, even when reviewing the same study.^26^ Such variation in use of waivers suggests the possibility of both over- and under-protection of participants. Furthermore, IRB members’ assessments may not align with prospective participants’ were it possible to ask them because, as noted previously, participants may see unanticipated ways in which the research affects them. Although community members’ views cannot substitute for IRB members’ considered judgments, such views may provide IRB members with greater insight into operationalizing regulatory requirements and may enhance their confidence in the ultimate judgment that waiving consent is (or is not) justified or that the trial is designed appropriately in the context of the planned waiver. Consultation might, for example, clarify whether the community agrees that risks associated with the proposed study are low.

Returning an earlier example, members of the IMPACT Lived Experience Panel expressed concern about interventions with the potential to disrupt or negatively affect their quality of life or to increase their dependence on others.^13^ When discussing a hypothetical trial looking at the effects of changes in staff shift length on residents in long-term care, panel members stated that interventions that reduced time spent with preferred caregivers would be “unwelcomed and disruptive.”^13^ Though the panel members were discussing a hypothetical trial, one can imagine similar feedback from community members alerting an IRB to risks or burdens to which they should attend. Reflecting on its consultation process, described briefly above, the COMPASS team described how the IRB maintained its oversight role and also learned from patient-stakeholders drawn from the population it was charged with protecting.^20^

## Implementing consultation

We propose that, by default, investigators considering conducting a PCT with a waiver of traditional consent should incorporate consultation with representatives of the affected community into the planning of their study. Rebutting the presumption in favor of consultation might involve a discussion of the context and nature of the intervention. As noted in the introduction, the Common Rule conditions eligibility for a waiver of consent (in part) on the research interventions being minimal risk; thus, risk is not a reliable signal of when consultation is valuable. Instead, we propose that the more research interventions affect care or the patient’s experience of care, the greater the requirement to seek consultation. This suggests that consultation would be more important for a study like TiME or COMPASS that materially shapes care delivery and relatively less important for a study that is purely educational in nature or that relies solely on record review.

When engaging patients or the public, there are at least four important questions.^27,28^ First, when in the course of designing a study should investigators pursue consultation? Second, whom should investigators consult? Third, how should the consultation process proceed? Fourth, should this be a regulatory requirement or an ethical recommendation?

### When to consult?

While there may be value in consultation at all stages of PCT design and conduct, community consultation intended to address the ethical challenges of waivers of traditional consent should occur early in the process of study development and design. That way, there is a genuine possibility of modifying the study, or even abandoning the plan to seek a waiver of consent, in response to feedback.

### Whom to consult?

In the United States, federal regulations allow some planned emergency research to be conducted with an exception from informed consent (EFIC) (21 CFR §50.24). In addition to meeting specific requirements regarding the study itself, investigators proposing an EFIC must consult “with representatives of the communities in which the clinical investigation will be conducted and from which subjects will be drawn” (21 CFR §50.24(a)(7)(i)). In the EFIC context, it is often hard to define the “community” with which to consult.^29^ The EFIC regulation defines the community both in geographic terms (i.e. where the investigation will be conducted) and in condition-focused terms (i.e. the potential population of participants). Defining the community for these studies has remained challenging, in part because emergency conditions are, by definition, episodic and because large portions of the community may be at risk.

By contrast, identifying the relevant community will often be more straightforward when an investigator proposes to conduct a PCT with a waiver. Patient-participants are typically enrolled at participating sites, often ones at which they already seek care, and many—though certainly not all—pragmatic trials target conditions that participants already have. For example, Implementation of a Randomized Controlled Trial to Improve Treatment With Oral Anticoagulants in Patients With Atrial Fibrillation (IMPACT-Afib) was a randomized PCT exploring whether an educational intervention targeted at patients with atrial fibrillation and their clinicians could promote appropriate use of oral anticoagulants and, in turn, prevent avoidable strokes.^30^ Five health plans worked with the IMPACT-Afib team to identify approximately 80,000 plan members with untreated atrial fibrillation. For purposes of consultation, the community and its membership were well-defined. Even when the community of potential participants is not so readily identifiable, it may be possible to identify patients recently treated for the condition of interest (e.g. stroke patients in COMPASS) or in the setting of interest (e.g. ICU patients in SUNSET ICU) and to ask for their input.

Once the relevant community is defined, investigators should approach consultation genuinely and try to seek out individuals within that community who will constructively engage with them—not individuals who simply accept the research team’s perspective or unquestioningly approve its agenda.^27^ Consultation doesn’t require community members to have any particular expertise or qualifications; what is needed is people who are interested and can provide thoughtful feedback.^31^ It may help to talk to clinicians and staff to get the names of individuals who listen well, respect others’ perspectives, and are comfortable interacting with different kinds of people. Or, there may be an existing community advisory board, patient and family advisory board, or patient advocacy group that the researcher can leverage, rather than identifying community members on their own.

Purposive selection is also important. Investigators should seek to engage with individuals with diverse backgrounds, experiences, and viewpoints. When relevant, this includes representation of a range of views on known areas of disagreement, lest the investigators engage with only a subset of the relevant community.^32^ For example, investigators conducting a PCT relevant to autism might, depending on the nature of the intervention, seek out the views of individuals across the autism spectrum—or, as appropriate, their caregivers—given vigorous debate regarding whether autism is a disorder or a difference.^33^ Investigators should also pay special attention to consulting with members of marginalized groups.

### How to consult?

There are many possible methods of consultation. We do not seek to be prescriptive. Rather, it is important for research teams to think creatively about the goals of consultation and key questions raised by their specific PCT. Then, consultation processes should be designed in a way that advances those goals. For instance, in prior work, one of us has engaged a patient advisory panel to design a patient-driven, context-appropriate consent process.^34^ A similar approach might be appropriate for some PCTs conducted with a waiver, while there may be other times when that is insufficient. For example, it might be possible to talk to an advisory panel as a first step and to pursue more focused engagement if substantive issues or specific concerns are raised. The extent of consultation that is likely to be helpful is related to the context and nature of the intervention.

Consultation methods may also be shaped by community members’ needs. For example, the IMPACT Lived Experience Panel was designed to have dementia-friendly modes of engagement.^35^ These included multiple meetings with ample time allotted for repetition and discussion, as well as the provision of simple written materials for pre-reading as well as for memorializing conversations.

The content of consultation will be trial specific, but when consultation is indicated because the research team is considering a waiver of traditional consent, there will be common themes. In light of the ethical challenges outlined above, an important part of any consultative process will be to explain the rationale for the proposed study and to speak to its value. It will be important to provide a justification for the requested waiver and to understand whether this justification is acceptable. Furthermore, investigators should use the consultation to identify ways that the study may be improved to enhance the benefits, to minimize risks or burdens, and to communicate with and treat participants respectfully.

### Is there an obligation to consult?

Consultation has been handled in different ways. As noted above, US regulations require that investigators proposing an EFIC trial consult with community representatives; the expectations for community consultation in this setting are high given the stakes in emergency care. By contrast, the Ottawa Statement recommends but does not require that, when interventions “may substantially affect cluster interests, researchers should seek to protect cluster interests through cluster consultation to inform study design, conduct, and reporting.”^3^ In addition, some studies in contexts other than PCTs have voluntarily made specific programmatic commitments to consultation with populations that have been historically mistreated in research.^36^

Our focus here has been to articulate the ethical value of consultation for PCTs involving waivers of consent and to suggest consultation should be the default, not to argue for a regulatory requirement that might devolve into a mere box-checking exercise or a routine expectation for expensive, labor-intensive efforts. While many forms of consultation require little in the way of resources, some studies may warrant greater efforts, and we encourage funders to recognize and support this investment. We also encourage IRBs to recognize the value of consultation; consultation does not undermine their oversight role but rather can inform how they fulfill it, especially when community input challenges traditional assumptions, for instance, about communication materials. Consultation can reflect a genuine spirit of partnership and collaboration, even in situations where informed consent is not practicable.

## Possible objections

First, some might object to the default that consultation is needed when investigators propose to seek a waiver of traditional consent and instead favor a rebuttable presumption that consultation *is not* needed. This, however, would effectively reinforce the status quo. Some, though not all, of the ethical challenges identified above are premised on the idea that investigators cannot know at the outset when a study—or a waiver—will or will not be acceptable to the affected community; a default in favor of no consultation ignores this concern.

Second, it is possible that some investigators will perform consultation in ways that are largely performative, lending a veneer of acceptability to a waiver that would not in fact be acceptable to members of the affected community. Yet, the fact that there are some bad actors should not close our minds to the virtues of consultation or to the importance of establishing a norm that engagement with participant communities is respectful and valuable even when seeking individual consent is not practicable.

Third, people may worry that consultation is burdensome and so will be a barrier to some research. We recognize that research teams conducting EFIC research often use public forums, meetings with community groups, focus groups, and surveys.^37^ The Ottawa Statement suggests similar mechanisms, including “open public fora, community advisory boards, meetings with opinion leaders, presentations at religious or civic organizations, and the use of radio, television, or the Internet.”^3^ These methods have faced criticism for being labor-intensive and expensive without generating actionable recommendations to inform study design.^38^ As described above, what we are recommending need not be burdensome. The key is to focus on ways to solicit meaningful input, focusing on the quality of interaction rather than the quantity of community members engaged. Efforts should involve substantive engagement of key stakeholders rather than broad sampling of populations. The nature and extent of the consultation effort should likely be scaled to the anticipated impact of a PCT on care. As a field, we’re still learning how to engage with participants well. There is value in studying the most efficient and meaningful approaches. We have outlined some of the goals of consultation; the extent to which these goals are realized with specific efforts needs further work. As we learn, it will be important to share these learnings and incorporate them into practice.

Fourth, we recognize that some community members, when consulted, may object to conducting the proposed (or any) study without prospective consent. This is not an argument against consultation. It is important to emphasize that those being consulted are not being asked for proxy consent nor are they being given veto power. Rather, they are being given an opportunity to learn about and give feedback on a proposed study, feedback that investigators can then weigh on a case-by-case basis. Experience suggests that some individuals who are ultimately enrolled in research using a waiver of consent would have preferred not to participate, and yet, waivers continue to be employed.^6^ Our fundamental argument is that, if a waiver is under consideration, it is better proactively to consult with participant communities than to proceed in the absence of such respectful and potentially informative engagement.

Fifth, we acknowledge that community consultation is likely to address only some concerns related to waivers of informed consent. For instance, research ethicists worry that the desire to waive consent may problematically drive—rather than being driven by—research design.^39^ Given that IRBs can struggle with identifying when this occurs, we think it’s unlikely that community members will be able to identify or address it. Our claim is not that consultation is a panacea for all waiver-related ethical concerns; our more modest claim is that, through input from community members, consultation helps address some of the ethical challenges that arise when individuals are enrolled in research using a waiver of informed consent.

## Conclusion

Although waiving traditional consent is often necessary in PCTs for logistical or methodological reasons, it is important to recognize the associated ethical challenges. By consulting with members of the target study population when planning and executing PCTs that propose to employ a waiver of consent, investigators can mitigate these challenges. Furthermore, because patient populations for PCTs are often well-defined and identifiable in advance, conducting consultations will usually be minimally burdensome and thus not a barrier to research. Given these likely advantages and minimal costs, consultation merits study as a way to enhance the ethics and practice of PCTs that propose to employ waivers.
